# Utilization of absorbance subtraction and ratio difference green spectrophotometric methods for the quantification of alfuzosin hydrochloride and tadalafil in their binary mixture

**DOI:** 10.1186/s13065-024-01201-7

**Published:** 2024-05-09

**Authors:** Ali Alqahtani, Taha Alqahtani, Sherif Ramzy

**Affiliations:** 1 Department of Pharmacology, College of Pharmacy, King Khalid University, Abha, 62529 Saudi Arabia; 2 Pharmaceutical Analytical Chemistry Department, Faculty of Pharmacy, Al-Azhar University, Cairo, 11751 Egypt

**Keywords:** Benign prostatic hyperplasia, Alfuzosin hydrochloride, Tadalafil, Absorbance subtraction, Ratio difference

## Abstract

Alfuzosin hydrochloride and tadalafil fixed-dose combination tablets were recently formulated for the treatment of individuals with lower urinary tract symptoms caused by benign prostatic hyperplasia. Herein, the first spectrophotometric methods for quantitative analysis of alfuzosin hydrochloride and tadalafil in their binary mixture were established. The spectral overlapping of alfuzosin hydrochloride and tadalafil made direct simultaneous analysis unfeasible. Therefore, two mathematical methods were used to solve these overlapping spectra: absorbance subtraction and ratio difference. The absorbance subtraction method manipulates the zero absorption spectra of the studied drugs at the isoabsorptive point (272 nm) and uses the absorbance factor of pure ALF to calculate the absorbance of the studied drugs in the mixture at the isoabsorptive point. The ratio spectra method, on the other hand, manipulates the ratio spectra of the studied drugs, which are obtained by dividing each drug’s zero absorption spectra by a divisor spectrum from the second drug. The ratio amplitude difference between 251 nm and 211 nm was directly proportional to alfuzosin hydrochloride, whereas between 292 nm and 222 nm it was directly proportional to tadalafil. The methods used were verified in accordance with the recommendations of the ICH and demonstrated adequate linear regression in working ranges of 1–15 µg/mL for alfuzosin hydrochloride and 3–40 µg/mL for tadalafil. The methods were accurate, precise, and selectively employed to quantify alfuzosin hydrochloride and tadalafil in their combined tablets.

## Introduction

**Fig. 1:**
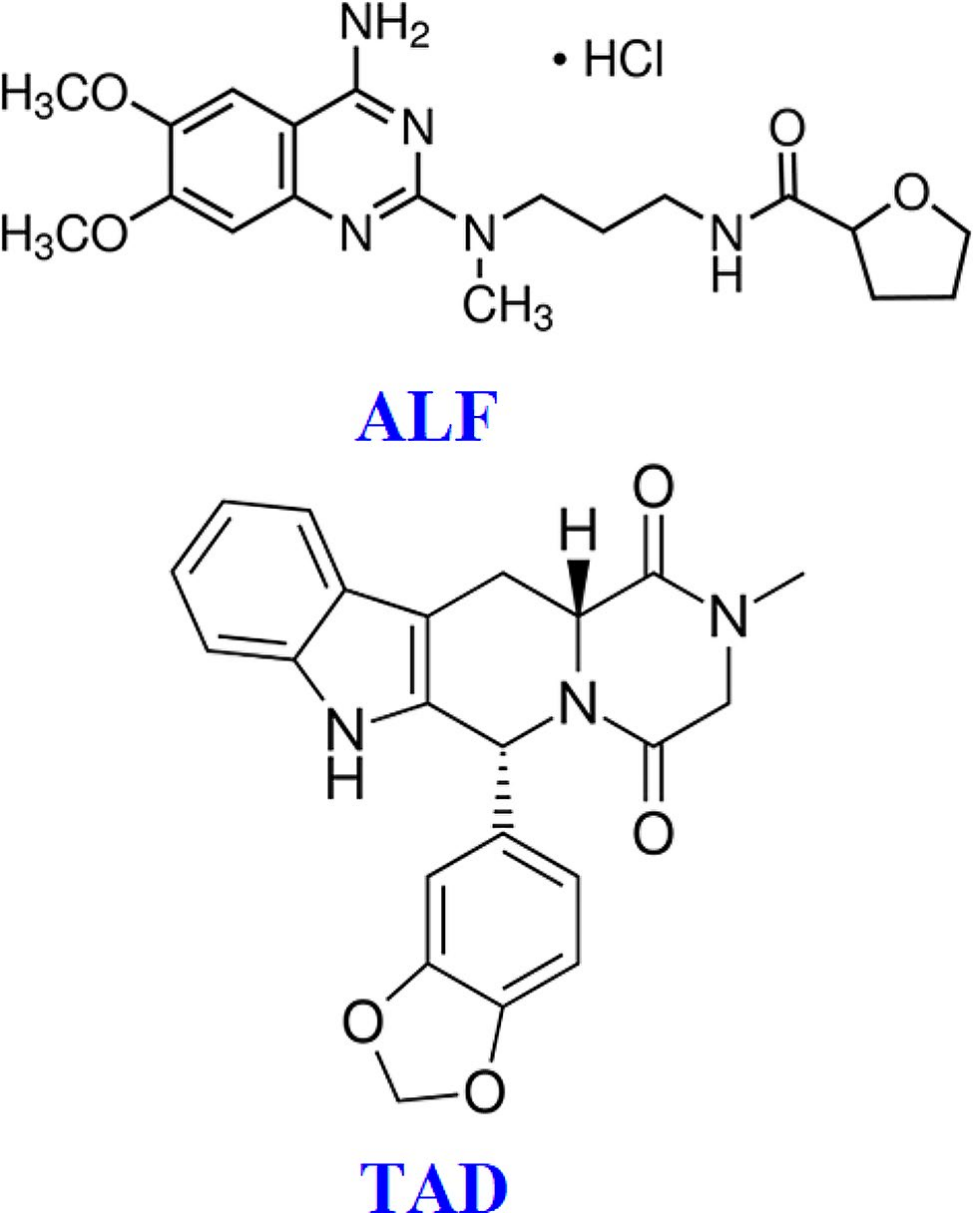
ALF and TAD chemical structures

Benign prostatic hyperplasia affects many elderly men and is accompanied by lower urinary tract symptoms and erectile dysfunction [1, 2]. Combination treatment with a selective alpha-1 blocker and a phosphodiesterase 5 inhibitor could significantly improve lower urinary tract symptoms and sexual function in patients [3, 4]. Alfuzosin hydrochloride (ALF), Fig. 1, is a second-generation uroselective alpha-1 adrenoreceptor inhibitor used for improving the symptoms of benign prostatic hyperplasia [5]. It selectively and competitively inhibits alpha-1 adrenoreceptors located in the prostate, bladder, and urethra, causing smooth muscle relaxation, and improving lower urinary tract symptoms and urine flow [6]. Tadalafil (TAD), shown in Fig. 1, is a phosphodiesterase 5 inhibitor that is used to treat erectile dysfunction and benign prostatic hyperplasia [7]. It increases blood flow and improves lower urinary tract symptoms by modulating nitric oxide/cyclic guanosine monophosphate levels in the corpus cavernosum [8]. In comparison to monotherapy, the combination of ALF and TAD has a synergistic relaxant effect on the corpus cavernosum and considerably improves lower urinary tract symptoms and sexual performance [9–11].

Recently. ALF and TAD fixed-dose combination tablets have been formulated. Only chromatographic [12] and synchronous spectrofluorometric [13] approaches for simultaneous detection of ALF and TAD were found in the literature. So far, no spectrophotometric approach for simultaneously determining ALF and TAD in their combination has been published.

The goal of this research was to provide the first spectrophotometric methods for determining ALF and TAD in their combination. The developed methods were absorbance subtraction [14] and ratio difference [15, 16]. The absorbance subtraction method simply manipulates the zero absorption spectra using an isoabsorptive point (λ_iso_) and an absorbance factor, whereas the ratio difference method manipulates the ratio spectra. The methods were validated using ICH criteria and selectively employed for determining ALF and TAD in pharmaceutical tablets without interference. The developed spectrophotometric methods offer an advantage over the reported methods in terms of procedure simplicity and affordability.

### Theory of absorbance subtraction method

If a mixture contains two drugs, X and Y, with overlapping spectra that intersect at an isoabsorptive point, where Y has a greater extension than X, and X does not exhibit any absorbance (A2) at a different wavelength (λ2), the isoabsorptive point (λiso) may be utilized for the individual quantitative analysis of both X and Y within the mixture (X + Y). This determination can be achieved through the utilization of a mathematically derived factor specific to one of the components. Through a straightforward manipulation process, the absorbance values corresponding to X and Y can be separately obtained. Consequently, the concentration of each component can be determined using the regression equation based on the isoabsorptive point, eliminating the necessity for an additional analytical method.

The absorbance values for X and Y at the isoabsorptive point were determined through the utilization of the absorbance factor {Aiso / A2}, a fixed value specific to pure Y that signifies the mean ratio between the absorbance readings of varying concentrations of pure Y at the λiso (Aiso) compared to those at λ2 (A2).

1 $$\begin{array}{l} \varvec{A}\varvec{b}\varvec{s}\varvec{o}\varvec{r}\varvec{b}\varvec{a}\varvec{n}\varvec{c}\varvec{e}\,\varvec{o}\varvec{f}\,\varvec{Y}\,\varvec{i}\varvec{n}\,\varvec{t}\varvec{h}\varvec{e}\,\varvec{m}\varvec{i}\varvec{x}\varvec{t}\varvec{u}\varvec{r}\varvec{e}\,\varvec{a}\varvec{t}\,{}_{\varvec{i}\varvec{s}\varvec{o}}\\=\frac{\varvec{a}\varvec{b}\varvec{s}1}{\varvec{a}\varvec{b}\varvec{s}2}\times \varvec{a}\varvec{b}\varvec{s}{}_{2(\varvec{X}+\varvec{Y})}\end{array}$$

2 $$\begin{array}{l} \varvec{A}\varvec{b}\varvec{s}\varvec{o}\varvec{r}\varvec{b}\varvec{a}\varvec{n}\varvec{c}\varvec{e}\,\varvec{o}\varvec{f}\,\varvec{X}\,\varvec{i}\varvec{n}\,\varvec{t}\varvec{h}\varvec{e}\,\varvec{m}\varvec{i}\varvec{x}\varvec{t}\varvec{u}\varvec{r}\varvec{e}\,\varvec{a}\varvec{t}\,{}_{\varvec{i}\varvec{s}\varvec{o}}\\=\varvec{a}\varvec{b}\varvec{s}{}_{\varvec{i}\varvec{s}\varvec{o}(\varvec{X}+\varvec{Y})}-\frac{\varvec{a}\varvec{b}\varvec{s}1}{\varvec{a}\varvec{b}\varvec{s}2}\times \varvec{a}\varvec{b}\varvec{s}{}_{2(\varvec{X}+\varvec{Y})} \end{array}$$

Where, abs1, abs2 is the absorbance of pure Y at λiso and λ2;

abs1/abs2 is called the absorbance factor and abs λiso (X + Y) and abs λ2(X + Y) are the absorbance of the mixture at these wavelengths (λiso, λ2).

The quantification of individual X and Y components involves the utilization of isoabsorptive point regression equations, which are derived from the absorbance values of zero order curves for X and Y plotted against their respective concentrations at the isoabsorptive point (λiso).

## Experimental

### Materials

The standard for ALF and TAD were provided by EVA Pharma, Egypt. Laboratory manufactured tablets were prepared, with each tablet containing ALF 10 mg, TAD 5 mg, 20 mg of talc powder, 15 mg of maize starch, and 7 mg of magnesium stearate. HPLC grade Ethanol was procured from Sigma-Aldrich, located in Darmstadt, Germany.

### Instruments

All spectra were collected using a UV-Visible Spectrophotometer (Model-1800 Shimadzu, Japan).

### Solutions

Standard solutions of ALF and TAD with a specified final concentration of 100 µg/mL were meticulously and freshly prepared through the precise process of transferring precisely 10 milligrams of each drug powdered into two distinct 100-milliliter volumetric flasks, followed by the addition of 60 milliliters of ethanol. The solutions were then subjected to rigorous agitation, ensuring thorough mixing, before being topped up to the calibrated volume with additional ethanol, in order to achieve the desired concentration levels.

### Calibration curves

Eight calibration standards of ALF (1–15 µg/mL) and TAD (3–40 µg/mL) were separately prepared by diluting definite aliquots of their respective standard solutions with ethanol. The absorption spectra of these solutions were measured between 200 and 400 nm.

#### Absorbance subtraction method

The absorbance values of pure ALF and TAD at λ_iso_ (272 nm) were graphed against their respective concentrations to construct the calibration graphs and the regression equations were derived. The absorbance factor of pure ALF was computed at 272 nm and 330 nm (A_272_/A_330_) and used to determine the absorbance of ALF and TAD in the mixture at λ_iso_. The concentrations of the studied drugs in the mixture were then calculated using the corresponding regression equation at λ_iso_.

#### Ratio difference method

ALF ratio spectra were obtained by dividing ALF absorption spectra by 20 µg/mL TAD spectrum, whereas TAD ratio spectra were obtained by dividing TAD absorption spectra by 7 µg/mL ALF spectrum. The difference values between ALF ratio spectra amplitudes at 251 nm and 211 nm were computed and plotted against the matched ALF concentrations to generate the calibration curve and the regression equation. Similarly, the difference values between TAD ratio spectra amplitudes at 292 nm and 222 nm were computed and plotted against the matched TAD concentrations to generate the calibration curve and the regression equation.

### Synthetic mixed solutions analysis

Five synthetic mixed solutions were meticulously created to test the selectivity of the applied methods by incorporating different concentration ratios of ALF and TAD. This was achieved through the precise transfer of specific volumes of ALF, amounting to 50, 80, 100, 120, and 120 µg, alongside corresponding volumes of TAD, measuring 50, 40, 50, 30, and 60 µg, into a series of five volumetric flasks, each with a capacity of 10 mL. Subsequently, the total volume within each flask was carefully adjusted using ethanol. The absorption spectra of these solutions were measured between 200 and 400 nm.

#### Absorbance subtraction method

The absorbance values of ALF in the mixtures at λ_iso_ (272 nm) were calculated using absorbance factor. The absorbance values related to TAD in the mixtures at λ_iso_ were then calculated by subtracting the absorbance values of the mixtures at λ_iso_ from the calculated absorbance values of ALF. The concentration of each drug was then calculated using the corresponding regression equation at λ_iso_.

#### Ratio difference method

The absorbance spectra of the mixture were divided by the divisor spectrum of ALF and TAD to get the ratio spectra. The difference values between 251 nm and 211 nm and between 292 nm and 222 nm were used to calculate the concentrations of ALF and TAD, respectively, using the corresponding regression equations.

### Pharmaceutical tablets analysis

Ten laboratory manufactured tablets, each comprising ALF 10 mg and TAD 5 mg, were powdered after being weighed. A quantity of powder equivalent to one tablet was dissolved in 50 mL ethanol, vortexed, and filtered into a 100-mL volumetric flask, and the volume was adjusted with ethanol. Five working samples of different concentrations were prepared by further dilution with ethanol. The samples were analyzed using the procedure described under synthetic mixed solutions analysis.

## Results and discussion

### Method development

**Fig. 2:**
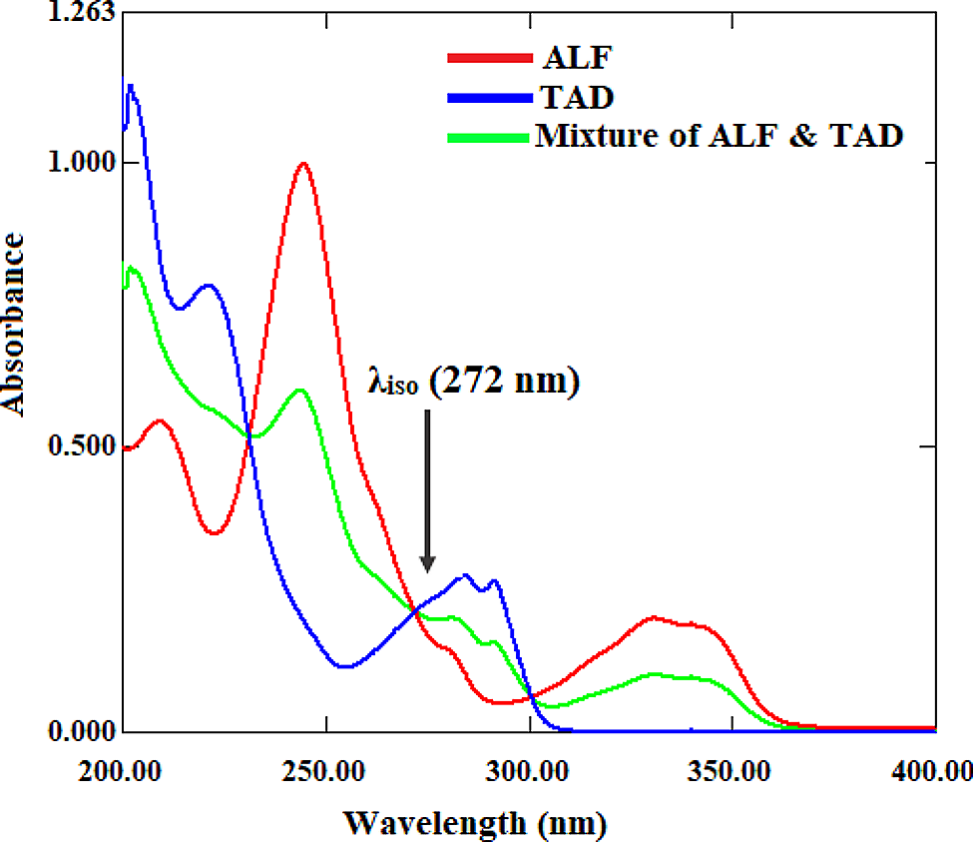
Absorption spectra of ALF (10 µg/mL), TAD (10 µg/mL), and their mixture (5 + 5 µg/mL)

The goal of this study was to develop straightforward spectrophotometric methods for determining ALF and TAD in their fixed-dose combination tablets. In the wavelength range of 200–310 nm, the UV absorption spectra of ALF and TAD were completely overlapping, with the isoabsorptive point (λ_iso_) at 272 nm. Furthermore, TAD has no absorption responses in the wavelength range 310–400 nm, whereas ALF exhibits a significant extended absorption peak at 330 nm (Fig. 2). These spectral overlaps make direct simultaneous determination of the studied drugs difficult. As a result, two mathematical methods, absorbance subtraction and ratio difference, were used to resolve the overlap and selectively quantify the studied drugs in synthetic mixed solutions and pharmaceutical tablets without interference.

#### Absorbance subtraction method

This method utilizes the isoabsorptive point (λ_iso_) at 272 nm for mathematical quantification of ALF and TAD in the mixture. The calibration curve for pure ALF and TAD at λ_iso_ were constructed against the corresponding drug concentrations, and the regression equations at λ_iso_ were derived. The absorbance factor for pure ALF was computed and used to determine the absorbance values of ALF and TAD in the mixture at λ_iso_. The absorbance factor for pure ALF (*A*_*ALF*_*at λ*_*iso*_*/A*_*ALF*_*at λ*_*2*_) was computed as the average of the ratio of absorbance values at 272 nm (λ_iso_) to those at 330 nm (λ_2_) of different ALF concentrations. The absorbances of ALF and TAD in the mixture were determined at λ_iso_ using the following two equations:

$$\begin{array}{l} Absorbance\,of\,ALF\,in\,the\,mixture\,at\,{\lambda }_{iso}\\= \frac{{A}_{ALF} at {\lambda }_{iso}}{{A}_{ALF} at {\lambda }_{2}} \times {A}_{Mix.} at {\lambda }_{2}\end{array}$$

$$\begin{array}{l} Absorbance\,of\,TAD\,in\,the\,mixture\,at {\lambda }_{iso}\\= {A}_{Mix. } at {\lambda }_{iso}- \left(\frac{{A}_{ALF} at {\lambda }_{iso}}{{A}_{ALF} at {\lambda }_{2}} \times {A}_{Mix.} at {\lambda }_{2}\right)\end{array}$$

Where *A*_*ALF*_ denotes the absorbance value of pure ALF and *A*_*Mix.*_ denotes the absorbance value of the mixture of ALF and TAD.

The determined absorbance values of ALF and TAD in the mixture at *λ*_*iso*_ were then used to calculate the concentration of each drug using the corresponding isoabsorptive point regression equation.

#### Ratio difference method

This method is based on the theoretical concept that dividing an absorption spectrum by a spectrum of the same substance yields a straight line parallel to the baseline. However, when the absorption spectrum of one molecule (X) is divided by the absorption spectrum of another compound (Y), a ratio spectrum is formed. The ratio spectrum’s difference in peak amplitudes between two specified wavelengths is proportional to the concentration of X without interference from Y.

**Fig. 3:**
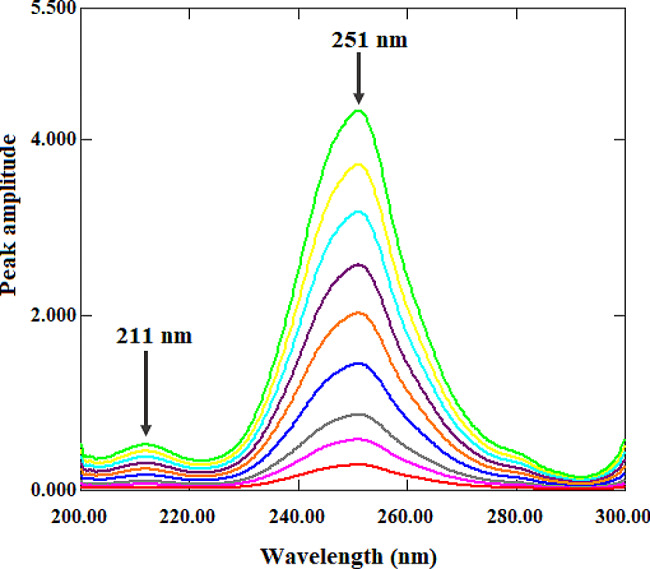
ALF ratio spectra (1–15 µg/mL) using TAD (20 µg/mL) as a divisor

**Fig. 4:**
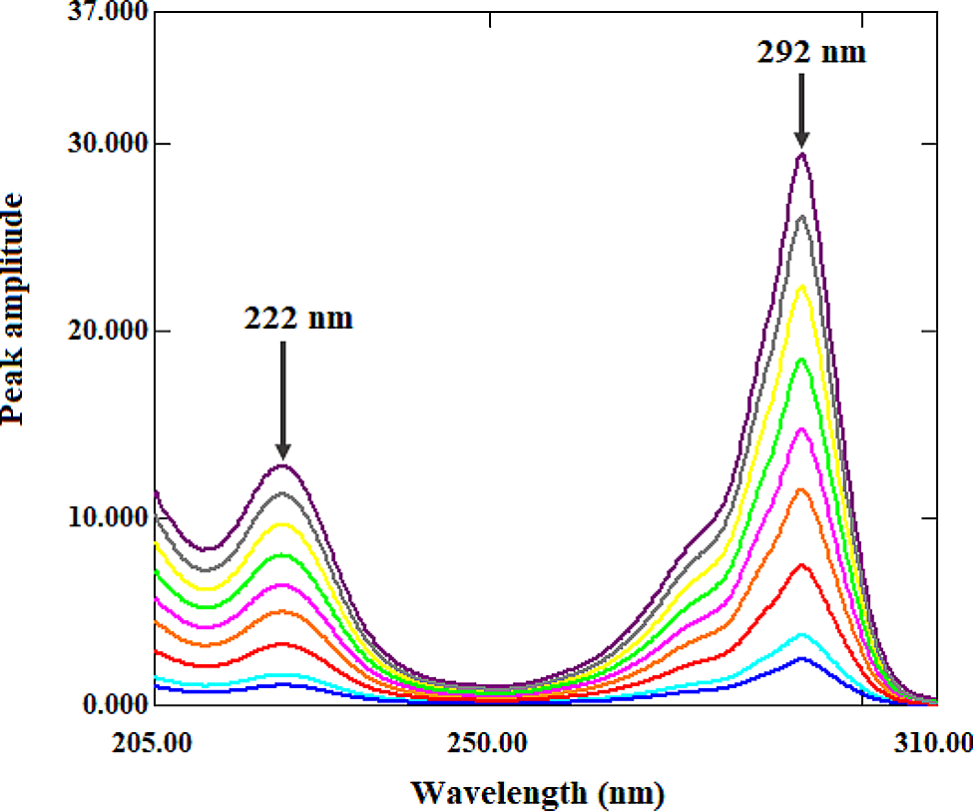
TAD ratio spectra (3–40 µg/mL) using ALF (7 µg/mL) as a divisor

The ratio spectra for ALF (Fig. 3) and TAD (Fig. 4) were obtained using this procedure by dividing the absorption spectra of each drug by an appropriate spectrum (divisor) of the second drug. This is an important step since the chosen divisor must strike a compromise between low noise and high sensitivity. Therefore, several ALF and TAD divisors were evaluated, and the best results were achieved with a 7 µg/mL ALF spectrum and a 20 µg/mL TAD spectrum. The ratio difference method calculates the difference in amplitude values of the ratio spectra at the two given wavelengths. As a result, it is critical to test the linearity at many wavelength pairs to select the wavelength pair with the best linearity and the lowest noise to signal ratio. The amplitude values of ALF ratio spectra at 251 nm and 211 nm were determined to have best linearity, and hence the difference between them was directly proportional to ALF with no interference from TAD. Further, the amplitude values of TAD ratio spectra at 292 nm and 222 nm were determined to have best linearity, and hence the difference between them was directly proportional to TAD with no interference from ALF. The calibration curve and regression equation were generated by graphing the difference in amplitude values at 251 nm and 211 nm for ALF and 292 nm and 222 nm for TAD against the corresponding ALF and TAD concentrations.

### Method validation and statistical comparison

The methods used were verified in accordance with ICH recommendations. Table 1 displays data on linearity range, regression equation parameters, accuracy, precision, as well as limits of detection (LOD) and quantitation (LOQ). The selectivity of the methods used was determined by their capacity to quantify the ALF and TAD in their synthetic mixed solutions and pharmaceutical tablets without interference from one another or tablet excipients. The results in Table 2 show that the methods used were able to selectively measure ALF and TAD in their synthetic mixed solutions without interference from one another. Furthermore, the results in Table 3 demonstrate that the employed methods were used to selectively quantify ALF and TAD in pharmaceutical tablets without interference from excipients, as proven by the results of standard addition technique. The t-test and F-test were used to do a statistical comparison between the outcome data gained by the applied methods and those obtained by the previously published method [12]. As indicated in Table 3, the data obtained demonstrate that no significant differences were discovered.

**Table 1:** Methods validation and regression data

Parameters	Absorbance subtraction		Ratio difference	
	ALF	TAD	ALF	TAD
Linearity range	1–15 µg/mL	3–40 µg/mL	1–15 µg/mL	3–40 µg/mL
Slope	0.0211	0.0211	0.2530	0.4165
Intercept	-0.0002	0.0035	0.0015	0.1064
Coefficient of determination (r^2^)	0.9999	0.9999	0.9999	0.9996
LOD	0.132 µg/mL	0.530 µg/mL	0.143 µg/mL	0.869 µg/mL
LOQ	0.401 µg/mL	1.606 µg/mL	0.432 µg/mL	2.633 µg/mL
Accuracy (%R)^a^	99.85	99.65	99.59	99.71
Repeatability precision (RSD)^b^	0.680	0.548	0.464	0.491
Intermediate precision (RSD)^b^	0.862	0.711	0.609	0.754

^a^ Mean of nine measurments (three concentration levels were repeated three times)

^b^ RSD of nine measurments (three concentration levels were repeated three times)

**Table 2:** The results of ALF and TAD synthetic mixed solution analysis

Taken (µg/mL)		%Recovery			
		Absorbance subtraction		Ratio difference	
ALF	TAD	ALF	TAD	ALF	TAD
5	5	99.10	98.72	99.25	98.90
8	4	100.31	99.96	99.68	99.56
10	5	100.42	99.36	99.82	100.58
12	3	99.22	100.26	99.13	100.98
12	6	99.01	98.73	98.96	99.78
Mean ± RSD		99.61 ± 0.694	99.41 ± 0.705	99.37 ± 0.370	99.96 ± 0.827

**Table 3:** Pharmaceutical tablets and standard addition procedure analytical findings, including statistical comparison

Parameters	%*R* ± RSD					
	Absorbance subtraction		Ratio difference		Reported method [12]	
	ALF	TAD	ALF	TAD	ALF	TAD
Pharmaceutical tablets (*n* = 5)	99.25 ± 0.662	100.03 ± 0.512	99.07 ± 0.755	99.66 ± 0.627	99.85 ± 0.936	100.12 ± 0.877
Standard addition (*n* = 3)	99.58 ± 0.598	99.35 ± 0.739	99.75 ± 0.668	99.90 ± 0.726		
t-test (2.306)^*^	1.168	0.183	1.460	0.951		
F-test (6.388)^*^	2.023	2.943	1.564	1.974		

^*^The values in parentheses are tabulated values of *t* and *F* at *P* = 0.05

## Conclusion

In this work, absorbance subtraction and ratio difference spectrophotometric methods were used to determine the concentrations of alfuzosin hydrochloride and tadalafil in their combination The methods were accurate, precise, and selectively employed to quantify alfuzosin hydrochloride and tadalafil in their combined tablets. The developed spectrophotometric methods offer an advantage over the reported methods in terms of procedure simplicity and affordability.

## Data Availability

No datasets were generated or analysed during the current study.

## References

[1] Calogero AE, Burgio G, Condorelli RA, Cannarella R, La Vignera S (2019). Epidemiology and risk factors of lower urinary tract symptoms/benign prostatic hyperplasia and erectile dysfunction. Aging Male.

[2] Glina S, Glina FPA (2013). Pathogenic mechanisms linking benign prostatic hyperplasia, lower urinary tract symptoms and erectile dysfunction. Ther Adv Urol.

[3] Kallidonis P (2020). Combination therapy with alpha-blocker and phosphodiesterase-5 inhibitor for improving lower urinary tract symptoms and erectile dysfunction in comparison with monotherapy: a systematic review and meta-analysis. Eur Urol Focus.

[4] Chen PC, Wang CC, Tu YK (2020). Combination alpha blocker and phosphodiesterase 5 inhibitor versus alpha-blocker monotherapy for lower urinary tract symptoms associated with benign prostate hyperplasia: a systematic review and meta-analysis. Urol Sci.

[5] Lee M (2023). Alfuzosin hydrochloride for the treatment of benign prostatic hyperplasia. Am J Health-Syst Pharm.

[6] Lefevre-Borg F (1993). Alfuzosin, a selective α1‐adrenoceptor antagonist in the lower urinary tract. Br J Pharmacol.

[7] Hatzimouratidis K (2014). A review of the use of tadalafil in the treatment of benign prostatic hyperplasia in men with and without erectile dysfunction. Ther Adv Urol.

[8] Yokoyama O (2015). Tadalafil for lower urinary tract symptoms secondary to benign prostatic hyperplasia: a review of clinical data in Asian men and an update on the mechanism of action. Ther Adv Urol.

[9] Oger S (2010). Combination of alfuzosin and tadalafil exerts an additive relaxant effect on human detrusor and prostatic tissues in vitro. Eur Urol.

[10] Oger S (2008). Combination of alfuzosin and tadalafil exerts in vitro an additive relaxant effect on human corpus cavernosum. J Sex Med.

[11] Kumar S, Kondareddy C, Ganesamoni R, Nanjappa B, Singh SK (2014). Randomized controlled trial to assess the efficacy of the combination therapy of alfuzosin and tadalafil in patients with lower urinary tract symptoms due to benign prostatic hyperplasia. LUTS: Lower Urinary Tract Symptoms.

[12] Surati J (2023). Validated thin-layer chromatographic–densitometric and high-performance liquid chromatographic methods for the simultaneous determination of alfuzosin and tadalafil in pharmaceutical products. JPC-J Planar Chromatogr.

[13] Elama HS, Shalan SM, El-Shabrawy Y, Eid MI, Zeid AM (2022). A synchronous spectrofluorometric technique for simultaneous detection of alfuzosin and tadalafil: applied to tablets and spiked biological samples. R Soc Open Sci.

[14] Lotfy HM (2014). Absorbance subtraction and amplitude modulation as novel spectrophotometric methods for the analysis of binary mixtures. Int J Pharm Pharm Sci.

[15] Batubara AS, Abdelazim AH, Almrasy AA, Gamal M, Ramzy S (2023). Quantitative analysis of two COVID-19 antiviral agents, favipiravir and remdesivir, in spiked human plasma using spectrophotometric methods; greenness evaluation. BMC chem.

[16] Abdelazim AH (2022). Quantitative Spectrophotometric Analysis of Celecoxib and Tramadol in their Multimodal Analgesia Combination tablets. J AOAC Int.

